# “Everyone Could Say What Was Inside”: emotional expression and interpersonal connection following emotion-focused awareness training

**DOI:** 10.3389/fpsyg.2026.1924332

**Published:** 2026-10-19

**Authors:** Deniz Akkaya Kozak, Gülüşýan Özgün Başýıbüyük

**Affiliations:** Department of Gerontology, Faculty of Health Sciences, Akdeniz University, Antalya, Türkiye

**Keywords:** emotion regulation, emotional awareness, emotion-focused awareness training, lifelong learning, older adults, qualitative research

## Abstract

Population aging has increased the need for interventions that support psychological wellbeing and emotional adaptation in later life. Although emotion-focused approaches have demonstrated promising outcomes, little is known about how older adults experience emotion-focused psychoeducational programs implemented within lifelong learning environments. This study explored older adults' experiences of participating in Emotion-Focused Awareness Training (EFAT), a group-based psychoeducational program integrating emotion-focused principles with EMDR-informed resourcing and stabilization techniques, delivered within a University of the Third Age. A qualitative descriptive design was employed. Semi-structured interviews were conducted with 56 older adults participating in a University of the Third Age lifelong learning program approximately 6weeks after completion of EFAT. Interview data were analyzed using thematic analysis. Participants described joining the program in search of emotional support, social connection, and opportunities for personal growth. They experienced EFAT as facilitating emotional awareness, emotional expression, psychological relief, stronger interpersonal relationships, and the integration of emotion-regulation strategies into everyday life, while also recognizing the value of a psychologically safe group environment for emotional learning. At the same time, some participants expressed a desire for greater individualized support, highlighting the inherent balance between shared learning and individual psychological needs within group-based psychoeducation. Overall, participants perceived EFAT as more than an educational program, describing it as an integrated psychosocial experience that supported emotional learning, interpersonal connectedness, and personal growth. These findings suggest that emotion-focused psychoeducational programs implemented within lifelong learning environments may represent a promising community-based approach to supporting emotional wellbeing, psychological adaptation, and healthy aging.

## 1 Introduction

Population aging represents one of the most significant demographic transformations of the twenty-first century, with the number of adults aged 60 years and older increasing rapidly worldwide (Cunningham et al., 2020; Tang et al., 2020; Gianfredi et al., 2025). Although increased life expectancy reflects major advances in public health and healthcare, it also highlights the importance of ensuring that longer lives are accompanied by psychological wellbeing and quality of life (Bar-Tur, 2021; Dogra et al., 2022). In recent years, psychological wellbeing has emerged as a key component of healthy aging and has been associated with lower mortality risk, better physical health, and greater life satisfaction (Kim et al., 2021a,b). Nevertheless, older adulthood is often characterized by significant life transitions, including retirement, bereavement, declining physical functioning, chronic health conditions, and changes in social relationships, all of which may challenge emotional adjustment and psychological functioning (Bar-Tur, 2021; Reynolds et al., 2022). These experiences can increase vulnerability to loneliness, social isolation, psychological distress, and reduced quality of life, particularly when adaptive coping resources are limited (Dogra et al., 2022; Gianfredi et al., 2025). Consequently, successful aging is increasingly understood not merely as the absence of disease, but as the capacity to adapt effectively to age-related changes and maintain psychological wellbeing despite adversity. Within this framework, resilience and emotion regulation are considered essential psychological resources that support emotional adaptation and promote positive aging outcomes (Ferrucci et al., 2020; Reynolds et al., 2022). Accordingly, understanding the psychological mechanisms that enable older adults to adapt to age-related challenges while maintaining psychological wellbeing has become a major priority in contemporary aging research.

Among the psychological mechanisms that support successful adaptation in later life, emotional functioning has received increasing attention. Nevertheless, such age-related transitions do not inevitably result in psychological distress. Rather, the impact of age-related challenges appears to depend largely on how individuals perceive, interpret, and regulate their emotional experiences (Adolfo et al., 2022). Within this framework, emotional awareness has emerged as a foundational psychological capacity that enables individuals to identify, differentiate, and make sense of their emotions. Greater emotional awareness has been associated with emotional clarity, adaptive coping, and psychological wellbeing, whereas difficulties in understanding emotional experiences have been linked to depressive symptoms, emotional vulnerability, and reduced wellbeing (Delhom et al., 2020; Meynadasy et al., 2022; Cho and Choi, 2024).

Closely related but conceptually distinct, emotion regulation refers to the processes by which individuals influence which emotions they have, when they have them, and how they experience and express them (Gross, 2015). While emotional awareness concerns the recognition and understanding of emotional states, emotion regulation encompasses the strategies individuals use to modify or manage those states in response to internal or external demands.

Importantly, emotional awareness is considered a prerequisite for effective emotion regulation, as individuals must first recognize and understand their emotional states before they can regulate them adaptively. Research suggests that older adults who demonstrate greater emotional awareness are more likely to employ adaptive regulatory strategies, such as acceptance, cognitive reappraisal, and emotional flexibility, while relying less on maladaptive strategies such as avoidance or expressive suppression (Tuicomepee et al., 2018; Delhom et al., 2020). Consistent with Socio emotional Selectivity Theory, older adults increasingly prioritize emotionally meaningful goals and seek to maintain positive emotional experiences through selective and adaptive regulatory processes (Isaacowitz, 2022; Thams and Brassen, 2023). These regulatory capacities have been consistently associated with resilience, life satisfaction, psychological wellbeing, and successful aging outcomes (Badawy et al., 2024; Kramer et al., 2026; Romera-Gasparico et al., 2026). Collectively, the literature suggests that emotional awareness and emotion regulation are not merely correlates of wellbeing but represent core psychological mechanisms through which older adults adapt to age-related challenges and maintain psychological health.

Although emotional awareness and emotion regulation are individual psychological resources, their development and application are strongly influenced by the social environments in which older adults live and interact. In this regard, lifelong learning has increasingly been recognized as more than an educational endeavor, functioning as an important psychosocial context that supports healthy and active aging. Organized lifelong learning environments, such as Universities of the Third Age (U3A), provide older adults with opportunities not only for continued learning but also for meaningful social participation, interpersonal connectedness, and engagement in purposeful activities (BuŽgová et al., 2024; Narushima et al., 2018; Rosser and Soler, 2024). These environments foster autonomy, competence, and social relatedness—three fundamental psychological needs identified by Self-Determination Theory as essential for optimal psychological functioning and wellbeing (Zhang et al., 2024). Consistent with this perspective, participation in lifelong learning programs has been associated with higher levels of psychological wellbeing, life satisfaction, resilience, and quality of life, while reducing loneliness, depressive symptoms, and the psychosocial consequences of age-related transitions (Bourassa et al., 2017; BuŽgová et al., 2024; Trică et al., 2024; Baczyńska and Musiał, 2025). Beyond their educational value, these settings provide opportunities for emotional exchange, mutual support, and continued personal growth, thereby strengthening older adults' capacity to adapt to the emotional and social challenges of later life (Narushima et al., 2018; Rosser and Soler, 2024). Consequently, lifelong learning environments represent particularly promising contexts for interventions designed to enhance emotional functioning, psychological wellbeing, and successful adaptation in older adulthood.

Emotion-focused approaches are grounded in the premise that emotions serve adaptive functions by providing essential information about individuals' needs, goals, and interactions with their environment. Accordingly, emotion-focused approaches—including Emotion-Focused Therapy (EFT) and emotion-focused psycho educational interventions—aim to enhance psychological functioning by increasing emotional awareness, facilitating adaptive emotional expression, and promoting more adaptive ways of understanding and regulating emotional experiences (Almeida et al., 2025; Pascual-Leone et al., 2025; Kramer et al., 2026). Accumulating evidence suggests that interventions targeting emotional awareness and emotion regulation can improve psychological wellbeing, resilience, quality of life, and emotional adjustment while reducing depressive symptoms and perceived stress among older adults (Badawy et al., 2024; Kramer et al., 2026; Romera-Gasparico et al., 2026). In particular, group-based formats appear to provide additional benefits by combining emotional skill development with opportunities for social interaction, mutual support, and shared emotional experiences, making them particularly well suited for emotion-focused psycho educational programs delivered within lifelong learning environments (Badawy et al., 2024; Perin et al., 2025).

However, existing emotion-focused interventions have predominantly been developed for clinical populations or therapeutic contexts, and structured psycho educational programs specifically designed to enhance emotional awareness and emotion regulation among community-dwelling older adults within lifelong learning settings remain scarce. Emotion-Focused Awareness Training (EFAT) addresses this gap by integrating emotion-focused principles with Eye Movement Desensitization and Reprocessing (EMDR) informed resourcing and stabilization techniques within a non-clinical, psycho educational framework tailored to the developmental needs and life circumstances of older adults. Unlike individual psychotherapy or clinically oriented group interventions, EFAT was specifically designed for delivery within lifelong learning environments, combining emotional skill development with the psychosocial benefits of group participation in a familiar community-based educational context.

Despite these promising findings, important gaps remain in the literature. Direct evidence for older-adult-specific emotion-focused interventions and structured emotion-focused psycho educational programs remains limited, with recent studies emphasizing the need for interventions specifically tailored to the emotional needs and developmental characteristics of later life (Almeida et al., 2025; d'Almeida and Cunha, 2026). Moreover, although community-based psychosocial interventions have demonstrated the feasibility of promoting emotional wellbeing outside clinical settings, emotion-focused interventions have rarely been implemented or evaluated within lifelong learning environments. This gap is particularly noteworthy because Universities of the Third Age and similar lifelong learning programs have consistently been associated with enhanced psychological wellbeing, social participation, and active aging, yet their potential as settings for emotion-focused interventions remains largely unexplored (BuŽgová et al., 2024). This gap highlights the need for structured emotion-focused psycho educational interventions specifically designed for older adults participating in lifelong learning programs.

To address these gaps, the present study developed and implemented EFAT, a novel group-based psycho educational program specifically designed for older adults participating in a lifelong learning program. Although lifelong learning environments are themselves associated with positive wellbeing outcomes, it remains an open question what structured emotion-focused psycho educational training adds to these benefits—particularly in terms of deepening emotional awareness, facilitating emotional expression, and equipping participants with transferable emotion-regulation strategies. The present study therefore positions its contribution not as an alternative to lifelong learning, but as an investigation of the added value of embedding structured emotion-focused programming within an existing lifelong learning context. The study aimed to explore how older adults experienced participation in EFAT, the meanings they attributed to the program, and how they integrated its emotional learning into their everyday lives. By providing an in-depth understanding of participants' experiences within an emotion-focused psychoeducational program delivered in a lifelong learning environment, this study contributes to the emerging literature on emotion-focused interventions in later life and highlights the potential of lifelong learning settings for fostering emotional wellbeing, emotional growth, and psychological adaptation. Specifically, the study addressed the following research question: How do older adults experience participation in EFAT, and what meanings do they attribute to this experience in the context of their emotional wellbeing and everyday lives?

## 2 Materials and methods

### 2.1 Study design

This qualitative study explored older adults' experiences of participating in the EFAT program implemented within a university-based lifelong learning setting. The study aimed to gain an in-depth understanding of how participants perceived the program, the meanings they attributed to their experiences, and the ways in which they integrated program-related learning into their everyday lives.

Semi-structured interviews were conducted following completion of the intervention to capture participants' subjective experiences and reflections on the training process. Interview data were analyzed using thematic analysis to identify recurring patterns of meaning across participants‘ experiences.

### 2.2 Participants and setting

Participants were older adults enrolled in Tazelenme University, a university-based lifelong learning program designed for adults aged 60 years and older in Türkiye. The present study included individuals who completed the EFAT program and subsequently participated in follow-up interviews exploring their experiences of the intervention.

A total of 56 participants were included in the qualitative component of the study. Participants ranged in age from 67 to 84 years (M = 72.18, SD = 4.01). Most participants were women (*n* = 49, 87.5%), while seven participants (12.5%) were men. This gender distribution is consistent with the broader demographic profile of the Tazelenme University lifelong learning program, in which female participants represent the majority of enrolled older adults, and therefore does not appear to reflect a selection bias specific to the EFAT intervention. Half of the participants held a university degree or higher (50.0%), and approximately two-thirds reported that their income was sufficient to meet their living expenses (69.1%). Most participants (67.9%) reported having at least one chronic health condition requiring regular medication use.

Participants were recruited from the intervention group of a psycho educational program conducted within Tazelenme University. Eligibility criteria included being aged 60 years or older, enrollment in Tazelenme University, regular attendance at the training sessions, and willingness to participate in a follow-up interview. Individuals with diagnosed psychotic disorders, dementia, Alzheimer's disease, or intellectual disabilities were not included in the program. Sixty-seven older adults began the EFAT intervention. Nine participants were excluded from the study due to insufficient attendance, defined as missing more than two of the ten sessions, and were therefore not eligible for the qualitative interviews. Of the 58 participants who completed the intervention, two declined to participate in the interviews. Consequently, the qualitative sample comprised 56 participants.

Tazelenme University provides educational, social, and developmental opportunities for older adults and aims to promote active aging, lifelong learning, and social participation. The EFAT program was implemented within this setting, allowing participants to engage in emotional awareness and emotion-regulation activities in a familiar educational and social environment.

### 2.3 Emotion-focused awareness training (EFAT)

EFAT is a structured group-based psycho educational program developed to enhance emotional awareness, emotional expression, and adaptive emotion-regulation skills among older adults. The program was designed for implementation within a lifelong learning setting and integrates principles from emotion-focused approaches together with EMDR-informed resourcing and stabilization techniques, including the safe place exercise, the four elements exercise, the container (box) technique, the butterfly hug, and the light stream exercise, as well as mindfulness-based breathing practices incorporated as a closing exercise in each session to support present-moment awareness and emotional regulation. Although EMDR was originally developed as a trauma-focused intervention, its resourcing and stabilization techniques — including the Safe Place Exercise, Butterfly Hug, and Container technique — have been increasingly recognized as versatile tools for enhancing emotional regulation, self-soothing, and internal resource development in non-trauma contexts (Korn, 2009; Hofmann et al., 2014). Within EFAT, these techniques were not employed for trauma processing but were adapted for psycho educational use to support participants in developing self-regulation capacities and strengthening their sense of internal safety, thereby complementing the emotion-focused framework of the program. The inclusion of EMDR-informed resourcing techniques in a non-clinical, community-based population is supported by evidence suggesting that stabilization and resourcing skills are beneficial not only for individuals with trauma histories but also for community-dwelling adults seeking to strengthen emotional self-regulation, enhance internal resources, and develop greater psychological resilience in everyday life (Korn, 2009; Hofmann et al., 2014). EFAT was delivered as a psycho educational intervention rather than a psychotherapy program.

The intervention consisted of 10 weekly sessions lasting approximately 90–120 min. Sessions combined brief psycho educational components with experiential exercises, reflective activities, guided group discussions, and structured home practice designed to encourage the application of emotional awareness and emotion-regulation skills in everyday life. Core topics included recognizing and labeling emotions, understanding the relationship between emotions, thoughts, and bodily experiences, developing empathy and emotional acceptance, differentiating emotional experiences, and strengthening adaptive emotion-regulation strategies.

Throughout the program, participants engaged in a range of experiential activities designed to increase emotional awareness and facilitate emotional expression. As the intervention progressed, greater emphasis was placed on emotional processing, self-reflection, and the application of emotion-regulation skills to everyday life. The final sessions focused on integrating acquired insights and reflecting on personal changes experienced throughout the program.

Table 1 provides an overview of the session structure, thematic focus, objectives, and key activities of the EFAT program.

**Table 1:** Overview of the EFAT session protocol.

Session	Thematic focus	Objectives	Key activities and techniques
1	Introduction to Emotional Awareness	Establishing group structure; developing foundational awareness of the role of emotions in daily life	Introductions and expectation setting; group rules and principles; psychoeducation on emotions; “Emotions in My Heart” drawing exercise (emotion mapping using colors)
2	Emotional Expression	Expanding emotional vocabulary; strengthening subjective emotional identification	Emotion vocabulary listing; “I-language” emotional self-description; Weekly Emotion Diary introduced as homework; Mindful Breathing exercise
3	Emotional Acceptance and Bodily Awareness	Increasing emotional and bodily awareness toward positive and negative emotions; developing acceptance and breathing/relaxation skills	Emotion Diary review; positive and negative emotion acceptance exercises using “I-language”; bodily awareness questions; psychoeducation on emotional diversity; Mindful Breathing exercise
4	Emotional Expression and Symbolization	Enhancing emotion recognition through body language; developing emotional expression and symbolization skills	Emotion Diary review; emotion enactment game (non-verbal emotion expression); Light Stream Exercise (EMDR-informed resourcing technique)
5	Empathy and Relational Awareness	Developing empathic listening and responsive communication skills	Mirror and Shadow activity (dyadic role-play); “Your Story, My Story” empathic listening exercise; Mindful Breathing exercise
6	Primary and Secondary Emotions	Identifying primary and secondary emotions in personal experience	Secondary emotion awareness activity; primary emotion identification and written exploration; Container/Box Exercise (EMDR-informed stabilization technique); homework: Genuine Emotions Form
7	Emotional Processing and Completion	Consolidating primary/secondary emotion awareness; addressing incomplete emotional processes	Genuine Emotions Form review; Empty Chair exercise (emotion-focused experiential technique; Greenberg and Watson, 2006); Mindful Breathing exercise
8	Emotional Schemas and Regulation	Identifying maladaptive emotional patterns; developing adaptive regulation strategies	“How Do I Cope?” group discussion; Safe Place Exercise with Butterfly Hug (EMDR-informed resourcing technique); homework: daily Safe Place practice
9	Positive Internal Dialogue	Strengthening positive self-talk and positive emotion; consolidating emotion regulation skills	Safe Place homework review; exploration of internal dialogues; “My Positive Quality” strengths-based enactment; Emotional Transformation activity (positive/negative emotion reframing); Mindful Breathing exercise
10	Integration and Closure	Reflecting on gains throughout the program; closing with positive emotions	Positive memory card review (homework debrief); “What Have I Gained?” reflection; Loving Bombardment activity (strengths affirmation in group); Mindful Breathing exercise; final evaluation

EMDR-informed techniques (Light Stream Exercise, Container/Box Exercise, Safe Place Exercise, Butterfly Hug) were adapted for psychoeducational use and delivered within a stabilization and resourcing framework, not as trauma-processing procedures. Detailed session materials, activity protocols, and the full intervention manual are not published in full to protect the intellectual rights of the EFAT program. The manual may be obtained from the corresponding author upon written request, subject to scientific and ethical use conditions. Mindful Breathing exercises were incorporated as a closing practice in each session to support present-moment awareness, emotional regulation, and session closure.

### 2.4 Data collection

Qualitative data were collected through semi-structured interviews conducted following completion of the EFAT program. Interviews were carried out during the follow-up assessment period approximately 6 weeks after the intervention had ended, allowing participants to reflect on both their immediate experiences and the perceived longer-term influence of the program.

An interview guide consisting of open-ended questions was developed to explore participants' expectations of the program, perceived benefits and limitations, experiences of specific activities, continued use of emotion-regulation strategies, and recommendations for future implementation. The semi-structured format allowed participants to elaborate on issues that they considered personally meaningful while ensuring consistency across interviews. The interview questions are presented in Table 2.

**Table 2:** Semi-structured interview guide.

Question	Focus area
What were your expectations of the psychoeducational program, and do you feel these expectations were met?	Program expectations and perceived outcomes
Among the various activities and exercises included in the program, which did you find most beneficial?	Activity evaluation
Were there any activities or exercises during the program that you did not enjoy or found challenging?	Negative experiences and difficulties
Are you able to apply what you learned during the program in your daily life?	Transfer of skills to everyday life
Do you have any suggestions for how the program could be improved?	Program evaluation and recommendations

Interviews were conducted using an open-ended, semi-structured format. Follow-up and probing questions were used flexibly in response to participants' individual narratives.

All interviews were conducted by the first author, a psychologist with doctoral training in Gerontology, who had also facilitated the intervention. Most interviews were conducted face-to-face; however, participants who were unable to attend the follow-up session because of health, transportation, or personal circumstances were interviewed by telephone. Interviews were conducted individually, audio-recorded with participants' consent, and lasted approximately 15–20 min. Audio recordings were transcribed verbatim prior to analysis. Rather than relying on *a priori* saturation criteria to determine sample size, interviews were conducted with all eligible participants who completed the EFAT program and consented to follow-up interviews, resulting in a relatively large and information-rich qualitative dataset. Although sample size in qualitative research is not determined by statistical power, the present sample is considered sufficient from an information power perspective (Malterud et al., 2016), given the specificity of the study aim, the use of a theory-driven analytical approach, and the dense, information-rich nature of the data generated through in-depth individual interviews with a well-defined participant group. To protect confidentiality, identifying information was removed from the transcripts, and participants were assigned numerical identifiers (e.g., P1, P2).

### 2.5 Data analysis

Interview transcripts were analyzed using thematic analysis (Braun and Clarke, 2006), with particular attention to inter-coder consistency through independent coding and reliability assessment. Data management and coding were conducted using MAXQDA 24 software.

The analytic process began with repeated reading of the interview transcripts to achieve familiarity with the data. Initial codes were generated inductively from participants' accounts and organized into meaningful categories. Through an iterative process of reviewing, comparing, and refining codes, broader themes were developed to capture shared experiences across participants. Themes were subsequently reviewed against the original transcripts to ensure that they accurately reflected participants' perspectives and the overall dataset. Finally, themes were defined, named, and supported with representative quotations.

This analytic process enabled the development of themes focused on emotional awareness, emotional expression, interpersonal connection, and emotion-regulation practices in later life.

### 2.6 Trustworthiness

Several strategies were employed to enhance the trustworthiness of the findings. First, all interviews were audio-recorded and transcribed verbatim to preserve participants' original accounts. Throughout the analytic process, themes were continuously compared against the interview transcripts to ensure consistency between interpretations and the source data.

To strengthen analytic rigor, an independent researcher holding a PhD in Social Work and experienced in qualitative research reviewed the coding structure and thematic organization. This researcher coded the transcripts independently and was blind to the primary researcher's coding decisions prior to comparison. Coding agreement between the primary researcher and the independent reviewer was examined using Cohen's kappa coefficient, calculated in SPSS across the full set of 56 coded transcripts. The analysis demonstrated excellent inter-coder reliability (κ = 0.981, *p* < 0.001). In cases where coding disagreements arose, the two researchers discussed the discrepant codes until consensus was reached through collaborative deliberation.

Credibility was further supported through the use of direct participant quotations, allowing readers to evaluate the relationship between the data and the interpretations presented. In addition, themes were repeatedly reviewed and refined throughout the analytic process to ensure that they accurately reflected participants' experiences and the overall dataset.

### 2.7 Reflexivity

Given the qualitative nature of this study, the researchers‘ roles and their potential influence on the research process were carefully considered throughout all stages of the study. The first author, a psychologist with doctoral training in Gerontology, a certified EMDR practitioner, and with prior experience in psycho educational interventions involving older adults, developed and facilitated the EFAT program and subsequently conducted all individual interviews. This close involvement with the intervention may have facilitated rapport and encouraged participants to share rich and detailed accounts of their experiences. At the same time, the researchers recognized that the first author's dual role as both facilitator and interviewer might have influenced participants' willingness to disclose negative experiences or express criticism of the program.

A related methodological concern is the potential influence of social desirability bias on participants' responses. Given that the interviewer was also the program facilitator, participants may have been inclined to present their experiences in a more favorable light, emphasizing positive outcomes while minimizing or omitting negative experiences or unmet expectations. This dynamic may have led to an overrepresentation of positive evaluations in the data, which should be taken into account when interpreting the findings. To minimize this potential influence, participants were informed before the interviews that all responses would remain confidential, would not affect their relationship with the researchers or the program, and that both positive and negative experiences were equally valuable to the study. During the interviews, the first author adopted a neutral, non-judgmental interviewing style and encouraged participants to discuss both the beneficial and challenging aspects of their experiences openly. To enhance the credibility of the analysis, all transcripts were independently coded by two researchers, coding discrepancies were resolved through discussion until consensus was reached, and inter-coder reliability was assessed using Cohen's kappa coefficient. Throughout data analysis, the researchers engaged in ongoing reflexive discussions to consider how their prior involvement with the intervention and their own perspectives might shape data interpretation.

### 2.8 Ethics statement

Ethical approval for the study was obtained from the Akdeniz University Faculty of Medicine Clinical Research Ethics Committee (Approval No. TBAEK-621, September 5, 2024). Institutional permission to conduct the study was granted by the Antalya Campus Coordination Office of Tazelenme University.

All participants received information about the purpose and procedures of the study and provided written informed consent prior to participation. Participants were informed that their involvement was voluntary and that they could withdraw from the study at any time without penalty. Permission to use all measurement instruments employed within the broader research project was obtained from the respective scale developers.

## 3 Results

### 3.1 Theme 1. Entering the program: seeking emotional relief, connection, and meaning

Participants entered Emotion-Focused Awareness Training with motivations that extended beyond learning, reflecting broader emotional, social, and developmental needs in later life. Their accounts suggested that participation was often driven by a desire to address emotional challenges, maintain social engagement, and continue personal growth.

Several participants described joining the program in the hope of improving their emotional wellbeing. Expectations frequently centered on managing anxiety, coping with distressing emotions, and gaining greater self-understanding. As one participant explained,

“I have some problems within myself, such as anxiety and sleep difficulties. I wondered whether I could overcome them” (P52).

Such accounts suggest that participants viewed the program as a potential source of emotional support and psychological strengthening.

The desire for social connection also emerged as a prominent motivation for participation. For some, attending the program provided a meaningful opportunity to remain active and engaged following retirement or during periods of increased time spent at home. One participant reflected,

“At least it gets us out of the house and keeps us active. After working for 37 years, I wondered how I would fill the emptiness that followed, and I thought perhaps I could fill it with this” (P7).

Similar motivations were expressed by participants seeking opportunities to maintain social relationships despite caregiving responsibilities and reduced social participation. As another participant noted,

“Our relationships with friends had always been very good, and I expected that to continue. Because of my spouse's illness, I spent most of my time at home, and I thought it would be beneficial for me to get out occasionally” (P20).

Participants also emphasized a desire for continued learning and self-development. Learning was often described not simply as acquiring information but as a way of remaining mentally active and engaged with life. As one participant stated,

“I wanted to learn things I did not know, revisit what I already knew, and keep my mind active” (P55).

Taken together, participants entered the program seeking emotional relief, social connection, and opportunities for continued growth.

### 3.2 Theme 2. From listening to speaking: emotional expression and psychological release

A central aspect of participants' experiences was the opportunity to express emotions that had previously remained unspoken, suppressed, or difficult to share with others. Participants did not describe emotional expression merely as talking about feelings; rather, they portrayed it as a process of acknowledgment, emotional release, and self-understanding. Within the group environment, emotional disclosure was frequently associated with feelings of safety, trust, and psychological relief.

For some participants, emotional disclosure extended beyond discussing everyday feelings and involved sharing experiences that had remained hidden for many years. One participant described revealing a deeply personal experience that had never previously been shared, despite prior efforts to address it through professional support:

“There was something I wrote about during the program that nobody had ever been able to get me to talk about, despite all the psychologists I had seen. I shared something that I had never told anyone before. It gave me a great sense of trust. Being able to get it out of me brought relief.” (P18)

Participants frequently linked emotional disclosure to a sense of relief and renewed self-awareness. Rather than passively receiving information, they described actively engaging with their emotions and reflecting on their personal experiences through interactions with others. As one participant explained:

“I had never felt this emotionally relieved before. Talking about our feelings felt like medicine. Once we began opening up, everyone started sharing what they truly felt. It was incredibly comforting. I reflected on who I was, what I needed to change, and how I could become a more understanding person. I realized that I had changed.” (P40)

Experiential activities appeared to play an important role in facilitating emotional awareness and expression. Participants frequently described exercises such as emotional journaling, emotion cards, and the empty-chair technique as opportunities to recognize and confront emotions that had previously remained outside conscious awareness. One participant described the empty-chair exercise as particularly powerful because

“people could not escape from their emotions” (P26).

Others reported discovering a broader range of emotions than they had previously recognized, suggesting that the activities expanded both emotional awareness and emotional vocabulary (P51).

Across interviews, emotional expression was consistently described as a meaningful and often transformative experience. Participants moved beyond simply carrying emotions internally and instead engaged in processes of sharing, reflecting on, and making sense of their emotional experiences.

### 3.3 Theme 3. Reconnecting with others: interpersonal closeness, trust, and group belonging

Participants frequently described the training as an experience that strengthened interpersonal relationships and fostered a sense of belonging. Although many participants had known one another through the university setting, they reported that the program enabled more meaningful interactions characterized by openness, trust, and mutual understanding.

A recurring theme across interviews was the development of closer social bonds through the sharing of personal experiences and emotions. Participants explained that discussing emotionally meaningful topics helped them get to know one another more deeply and encouraged interactions beyond the classroom setting. As one participant reflected:

“Although we had been attending classes together for a long time, we had never become this close. Through this training, we spent more time together and had more opportunities to get to know one another. After the sessions, we would sit somewhere and continue talking. We now have a group of ten friends who still meet regularly.” (P12)

Participants also described becoming more attentive to others' feelings and perspectives, which facilitated stronger interpersonal connections. One participant explained:

“I had the opportunity to get to know my friends more closely. I realized how important it is to understand people's emotions by looking into their eyes and communicating with them. I used to approach people more cautiously, but now it feels easier to connect with others.” (P51)

The group environment further encouraged participants to speak openly and feel accepted by others. As one participant noted:

“After the group activities, I think I overcame some of these difficulties. I realized that I should participate, express my opinions, and talk about myself.” (P56)

Similarly, another participant emphasized the value of mutual openness within the group:

“Getting to know one another felt good. We asked each other questions. Everyone was able to say what was inside them” (P22).

Overall, participants described the program as more than an educational experience. Through shared emotional experiences and ongoing interactions, participants developed interpersonal closeness, trust, and a sense of belonging that often extended beyond the duration of the program.

### 3.4 Theme 4. Learning to regulate emotions in everyday life

Participants frequently described drawing upon emotion-regulation strategies learned during the training when faced with everyday stressors, distressing emotions, or challenging life circumstances. Rather than remaining confined to the group setting, several techniques were incorporated into daily routines, suggesting that participants actively transferred aspects of the intervention into their personal lives.

Breathing exercises were among the most frequently used strategies. Participants described using these techniques to promote calmness, focus attention, and manage emotional discomfort. As one participant explained:

“I do it. I tell myself that the breath enters cold through my nose and leaves warmer. I focus on my breathing, send the breath to my heart, place my feet firmly on the ground, and tell myself not to pay attention to the sounds around me.” (P40)

Similarly, another participant described how breathing exercises gradually became integrated into everyday life:

“I do it sometimes. Breathing exercises are the ones I use most. I do not practice them every day, but whenever I go outside, I find myself wanting to do them. It is the exercise that became part of my life the most.” (P49)

Participants also reported adapting other emotion-regulation techniques to cope with unwanted thoughts and difficult emotions. Some described using symbolic exercises, such as the “box” technique or the “four elements” exercise, as practical tools for managing emotional experiences. As one participant noted:

“When I need it, I do it before going to bed or when I wake up. If something unpleasant happened the day before, I close the lid of the box again so that I do not carry it into the next day.” (P6)

Another participant similarly explained:

“I incorporated the four elements into my life. The exercises were very helpful. It felt as though the negative feelings inside me were disappearing.” (P51)

At the same time, participants' accounts suggested that sustaining these practices was not always straightforward. Caregiving responsibilities, competing daily demands, and difficulties remembering the techniques sometimes limited continued use. As one participant explained:

“I cannot say that I continued the exercises. My spouse is ill, and I spend most of my time taking care of them. I learned the techniques, but I often forget them.” (P30)

Taken together, participants described integrating a range of emotion-regulation strategies into their everyday lives, although the extent to which these practices were sustained varied across individuals.

### 3.5 Theme 5. Tensions and unmet needs within the group process

Although participants generally evaluated the training positively, some accounts highlighted limitations of the group-based format and unmet expectations regarding the intervention. These experiences primarily reflected preferences for more individualized support, greater personal feedback, and challenges associated with group dynamics.

Several participants expressed a desire for more individualized interaction and psychological guidance. While they valued the group experience, some expected opportunities for one-to-one conversations or personalized feedback regarding their emotional experiences. As one participant explained:

“I think it would have been more beneficial if there had been more one-to-one conversations. In many of the group activities, I felt a sense of pressure.” (P11)

Similarly, another participant described expecting more individualized feedback based on the activities completed during the program:

“I thought that after the activities we completed each week, we would talk about the results individually. If we discussed something upsetting, I expected guidance about what I could do.” (P19)

Others wished that the intervention had explored personal psychological experiences in greater depth. As one participant noted:

“We could have gone deeper into our psychology. There could have been individual sessions, more like seeing a psychologist.” (P34)

Participants also identified challenges related to group dynamics. In particular, some felt that dominant speakers occasionally limited opportunities for quieter individuals to contribute. One participant reflected:

“Some participants talked for a very long time, and those who spoke less did not always get a chance to contribute. A few times, I felt that I could not express the emotions I wanted to share because the discussion had already moved on.” (P43)

Taken together, participants varied in their expectations of the intervention and in the extent to which the group format met their individual needs.

## 4 Discussion

The present findings suggest that older adults' motivations for participating in EFAT extended well beyond an interest in learning about emotions. Participants described joining the program not only to acquire new knowledge but also to seek emotional support, maintain social connectedness, and continue their personal development while adapting to important life transitions such as retirement, caregiving responsibilities, and increased time spent at home. These findings are consistent with previous research demonstrating that participation in Universities of the Third Age and other lifelong learning programs is rarely driven by educational motives alone. Rather, older adults are motivated by broader psychosocial goals, including opportunities for meaningful social relationships, emotional wellbeing, purpose, and continued self-development (Formosa, 2021; Hachem, 2023; Yeo et al., 2026).

Although Self-Determination Theory did not inform the *a priori* design of the study or the interview guide, it emerged as a useful interpretive framework during the analysis phase, offering a theoretical lens through which to make sense of the patterns observed in participants' accounts. From a theoretical perspective, these findings can be interpreted through Self-Determination Theory, which proposes that psychological wellbeing is promoted when the basic psychological needs for autonomy, competence, and relatedness are supported (Tang et al., 2020). Participation in EFAT appeared to reflect all three needs: participants voluntarily engaged in the program because it addressed personally meaningful concerns (autonomy), viewed learning about emotions as an opportunity for continued growth and self-development (competence), and valued supportive interpersonal relationships within the group (relatedness). This interpretation is also consistent with Socio emotional Selectivity Theory, which suggests that older adults increasingly prioritize emotionally meaningful experiences and close social relationships as they age (Carstensen and Hershfield, 2021). More broadly, the present findings resonate with the introductory rationale of the study by suggesting that emotional awareness and emotion regulation—identified as core psychological resources supporting resilience and positive aging outcomes—were not only targeted by EFAT as program objectives but were also experienced by participants as personally meaningful and practically applicable in their everyday lives, thereby supporting emotional adaptation in later life.

Viewed together, these findings suggest that the appeal of EFAT was not solely attributable to its emotion-focused content but also to its congruence with the broader psychosocial motivations underlying participation in lifelong learning. In this respect, EFAT appears to function as more than a psycho educational program; it provided a context in which emotional learning, interpersonal connection, and personal growth were experienced simultaneously. These findings extend the current literature by suggesting that lifelong learning environments may represent particularly valuable contexts for implementing emotion-focused psycho educational programs that support emotional adaptation and psychological wellbeing in later life.

Participants' accounts indicate that emotional expression during EFAT involved considerably more than verbalizing feelings. Instead, emotional disclosure was experienced as a dynamic process through which increased emotional awareness facilitated deeper reflection, emotional processing, greater self-understanding, and, ultimately, psychological relief. This interpretation is consistent with contemporary literature, which emphasizes emotional awareness as a prerequisite for emotional processing, suggesting that individuals must first recognize and tolerate their emotional experiences before they can meaningfully express, process, and integrate them (Gray et al., 2021; Isaacowitz, 2022). Similarly, research on emotional disclosure suggests that sharing previously unspoken emotional experiences promotes psychological relief by facilitating reflection, cognitive integration, and meaning-making, rather than simply providing emotional release (Croes et al., 2024; Ho et al., 2018; Luo and Hancock, 2020).

Another important finding concerns the role of trust and psychological safety in facilitating emotional disclosure. Participants repeatedly emphasized that the group environment enabled them to share experiences they had never previously disclosed, even in professional settings. This finding suggests that the supportive interpersonal climate established throughout EFAT reduced the perceived risks associated with emotional disclosure and encouraged participants to engage more openly with emotionally significant experiences. This interpretation is consistent with evidence identifying psychological safety as a fundamental condition for meaningful emotional disclosure, particularly when individuals disclose experiences that have long remained suppressed (Hunt et al., 2021). Recent studies further indicate that structured group-based psycho educational programs can foster psychologically safe environments by providing opportunities for reflection, mutual support, and emotionally validating interactions, even outside formal psychotherapeutic settings (Sawyer et al., 2023; Aragonès et al., 2026).

Overall, these findings suggest that the value of EFAT extended beyond providing opportunities for emotional expression alone. Rather, the program appeared to facilitate a structured process in which emotional awareness evolved into emotional disclosure, emotional processing, and greater self-understanding within a psychologically safe group environment. In this respect, EFAT functioned not only as a psycho educational intervention but also as a supportive relational context that promoted emotional learning, interpersonal connectedness, and personal growth. The present findings therefore extend the existing literature by demonstrating that emotionally meaningful disclosure and emotional processing can emerge within a structured psycho educational program implemented in a lifelong learning environment, without requiring a formal psychotherapeutic framework.

An important interpersonal outcome of EFAT was the development of stronger and more meaningful relationships among participants. Although many participants had known one another through the University of the Third Age prior to the program, they described developing deeper interpersonal relationships characterized by greater trust, openness, empathy, and mutual understanding. These findings suggest that shared emotional experiences transformed the quality of existing relationships rather than simply creating new social contacts. This interpretation is consistent with evidence indicating that group-based interventions are most effective when they foster emotionally meaningful interaction, reciprocity, and interpersonal trust rather than merely increasing the frequency of social contact (Suriyawong et al., 2025).

Participants' accounts further suggest that emotional sharing promoted a stronger sense of belonging by encouraging empathic listening, mutual validation, and sustained interpersonal engagement beyond the structured sessions. Contemporary literature similarly emphasizes that relationship quality and belonging are more strongly associated with psychological wellbeing in later life than the size of one's social network, highlighting the importance of emotionally meaningful interpersonal connections (Michalski et al., 2020; Manchana, 2023). This interpretation is further supported by evidence indicating that structured group programs strengthen interpersonal relationships when they foster trust, mutual support, shared purpose, and emotionally meaningful interactions, thereby promoting a stronger sense of belonging among older adults (Simionato et al., 2023; Suriyawong et al., 2025).

Importantly, the present findings extend previous research on lifelong learning by suggesting that the social value of University of the Third Age programs lies not only in promoting participation but also in enriching the emotional quality of interpersonal relationships. Rather than functioning solely as an educational group, EFAT appeared to create a relational environment in which emotional openness, shared emotional reflection, and mutual understanding deepened existing interpersonal relationships and fostered enduring experiences of belonging and interpersonal connectedness.

An important practical outcome of EFAT was participants' ability to incorporate emotion-regulation strategies into their everyday lives. Rather than remaining confined to the intervention setting, techniques such as breathing exercises, imagery-based exercises, and symbolic emotion-regulation strategies became practical emotion-regulation tools that participants used to manage daily stressors and emotionally challenging situations. This interpretation is consistent with evidence indicating that emotion-regulation interventions are designed not only to enhance emotional functioning during sessions but also to facilitate the transfer of learned skills into everyday life through repeated practice and between-session application (Lawlor et al., 2022; Moore et al., 2022). Recent evidence also suggests that psycho educational interventions can promote emotional wellbeing by equipping participants with practical self-regulation strategies that can be applied beyond the intervention setting (ElKholy et al., 2024).

Participants' accounts also demonstrated that sustaining these strategies was influenced by the realities of everyday life. Caregiving responsibilities, competing daily demands, and difficulties remembering specific techniques sometimes limited their continued use. These findings are consistent with previous research suggesting that maintaining newly acquired self-regulation skills after brief psycho educational interventions is often challenging, particularly when everyday demands reduce opportunities for continued practice. Evidence further indicates that the long-term maintenance of intervention gains is facilitated by ongoing rehearsal, contextual support, and opportunities for reinforcement following the completion of structured programs (Moore et al., 2022; Aragonès et al., 2026).

Considered together, these findings suggest that EFAT facilitated the meaningful transfer of emotion-regulation skills into participants' everyday lives. Although participants differed in the extent to which they sustained individual techniques, the findings indicate that the strategies introduced during EFAT were perceived as sufficiently meaningful and practical to be incorporated into everyday emotional coping. At the same time, the variability in continued practice underscores the importance of follow-up opportunities for practice and reinforcement to support the long-term maintenance of newly acquired emotion-regulation skills.

Although participants evaluated EFAT positively overall, some also described unmet individual needs that reflected the inherent limitations of a group-based psycho educational format. Some participants expressed a desire for more individualized feedback, occasional one-to-one discussions, or greater opportunities to explore their own emotional experiences. These findings suggest that participants differed in the extent to which they expected individualized exploration of their emotional experiences within a group-based intervention. This interpretation is consistent with evidence indicating that group-based interventions for older adults are generally effective but cannot fully accommodate the diverse needs and preferences of every participant (Giebel et al., 2022; Romera-Gasparico et al., 2026).

Participants also identified challenges related to group dynamics, particularly when more dominant members limited opportunities for quieter participants to contribute. These findings reinforce the importance of active facilitation in maintaining balanced participation and psychologically supportive group processes. Contemporary implementation research suggests that tailoring interventions to participants' needs, encouraging equitable participation, and incorporating opportunities for individualized support can enhance both the acceptability and effectiveness of psycho educational programs for older adults (Riadi et al., 2022; Suriyawong et al., 2025; Tao et al., 2026).

Taken together, these findings suggest that the unmet individual needs identified by some participants reflect the inherent tension that group-based psycho educational interventions must achieve between shared learning and individualized support. While the strengths of group-based psycho education lie in fostering emotional normalization, peer learning, and interpersonal support, individualized exploration is necessarily more limited. Future implementations of EFAT may therefore benefit from incorporating optional individual feedback opportunities, explicit strategies to promote balanced group participation, and follow-up support to better accommodate participants with differing emotional needs while preserving the advantages of the group format.

Viewed collectively, the five themes suggest that EFAT functioned as more than an emotion-focused psycho educational program. Implemented within a University of the Third Age, the program provided an integrated psychosocial context in which emotional learning, interpersonal connectedness, and everyday emotion regulation developed through shared participation and reflection. Rather than operating solely as an educational intervention, EFAT appeared to facilitate multiple interconnected processes, including emotional awareness, emotional expression, psychological relief, stronger interpersonal relationships, and the application of emotion-regulation strategies in everyday life. At the same time, participants' accounts highlighted that, while group-based psycho education provides valuable opportunities for emotional learning and peer support, it cannot fully address every participant's need for individualized psychological guidance.

It is also noteworthy that participants repeatedly described their experiences within EFAT in terms of personally meaningful activities, interactions, and opportunities for growth. Although the present study did not explicitly target meaning in life as an outcome, these accounts suggest that emotion-focused psycho educational programs may also engage participants' sense of meaning—a dimension of psychological wellbeing that has received growing attention in the gerontological literature (**?**). Future research may benefit from explicitly examining whether and how emotion-focused interventions contribute to meaning-making processes in later life. At a practical and translational level, the present findings have direct implications for the design and delivery of psycho educational programming within lifelong learning settings. Specifically, the integration of EFAT into U3A curricula would entail offering structured, time-limited group sessions facilitated by a trained professional with expertise in gerontology and psycho educational intervention—such as gerontologists, psychologists, or other qualified professionals working with older adult populations, delivered within the existing organizational infrastructure of the program. Given that U3A settings already provide an established social network, a shared educational purpose, and a non-stigmatizing environment, they represent particularly suitable venues for embedding emotion-focused programming as a complementary offering alongside existing educational and social activities. Program coordinators may consider EFAT as a structured psychosocial enrichment module that does not require participants to identify as having mental health needs, thereby reducing barriers to engagement and broadening accessibility among older adults who might not otherwise seek psychological support. More broadly, these findings reinforce the growing recognition that lifelong learning environments can serve not only as educational settings but also as important community-based contexts for promoting emotional wellbeing, social participation, and healthy aging (BuŽgová et al., 2024). It is worth noting that lifelong learning environments such as Universities of the Third Age are distinct from generic community-based settings in several important respects. They provide a structured educational framework, an established social infrastructure, and a shared sense of purpose among participants, all of which may facilitate the implementation and uptake of psycho educational programs in ways that less structured community settings may not. At the same time, this distinction implies that the findings of the present study may not be directly transferable to other community-based contexts where such pre-existing structures and social bonds are absent. In this respect, EFAT appears to occupy an important position at the intersection of education, psychosocial support, and preventive mental health. By integrating emotion-focused learning within an existing lifelong learning program, the present study extends the literature by demonstrating how community-based educational settings can support emotional adaptation, strengthen interpersonal resources, and promote psychological wellbeing among older adults without relying on a formal psychotherapeutic framework. Collectively, these findings suggest that emotion-focused psycho educational programs implemented within lifelong learning environments may represent a promising community-based approach for supporting healthy aging by integrating emotional development with continued learning and social participation. Nevertheless, the present findings also underscore that group-based psycho educational formats are not sufficient to meet every participant's need for individualized psychological support, and that the sustainability of emotion-regulation skills beyond the intervention period remains a challenge that warrants attention in future program development and implementation.

### 4.1 Strengths and limitations

One of the major strengths of the present study is that it provides, to our knowledge, the first qualitative evaluation of EFAT, a novel emotion-focused psychoeducational program integrating emotion-focused principles with EMDR-informed resourcing and stabilization techniques, specifically developed for older adults and implemented within a University of the Third Age. By exploring older adults' lived experiences following participation in EFAT, the study extends beyond evaluating the intervention itself and provides insight into how emotional awareness, emotional expression, interpersonal connectedness, and emotion-regulation skills are experienced and integrated into everyday life within a lifelong learning context. The relatively large qualitative sample, excellent inter-coder reliability, and the inclusion of rich participant quotations strengthen the credibility and trustworthiness of the findings. Notably, rather than employing *a priori* saturation-based sampling, the study included all eligible participants who completed the intervention, an approach that minimized selection bias and ensured that the perspectives of the full completer cohort, rather than a purposively selected subset, were represented in the analysis. Furthermore, implementing EFAT within an established lifelong learning program enhances the ecological validity of the study by demonstrating how emotion-focused psycho education can be delivered in a real-world community setting.

Several limitations should also be acknowledged. First, participants were recruited from a single University of the Third Age, which may limit the transferability of the findings to other lifelong learning contexts or cultural settings. Second, the sample consisted predominantly of women, reflecting the demographic characteristics of the program but limiting the extent to which the findings represent the experiences of older men. Third, the absence of assessor blinding represents a methodological limitation, as the researcher who facilitated the intervention also conducted all interviews. This dual role may have introduced observer bias and influenced participants‘ willingness to disclose negative experiences or critical evaluations of the program, despite the measures taken to encourage open and honest responses. Fourth, interviews were conducted approximately 6 weeks after the intervention; therefore, the long-term sustainability of participants' experiences and perceived changes could not be explored. Fifth, participants were enrolled in an established lifelong learning program and may have already benefited from the psychosocial resources inherent to that setting, including opportunities for social participation, emotional exchange, and mutual support. It is therefore difficult to determine the extent to which the observed experiences reflect the specific effects of EFAT as an intervention, as opposed to the broader psychosocial context of the lifelong learning environment itself.

Sixth, many participants had prior acquaintance with one another through the lifelong learning program before the commencement of EFAT. Pre-existing social relationships may have facilitated the development of trust and psychological safety within the group, and it remains unclear whether similar dynamics would emerge in groups composed of participants who do not share an established social context. The generalizability of the findings to open or newly formed groups should therefore be considered with caution.

Seventh, the present study did not systematically examine whether participants' experiences or perceived benefits differed as a function of gender. Given the predominance of women in the sample, potential gender differences in responses to emotion-focused psycho education warrant further investigation in future research with more balanced samples.

Eighth, although Self-Determination Theory provided a valuable framework for understanding the psychosocial context of the lifelong learning environment, it remains unclear whether the fulfillment of autonomy, competence, and social relatedness needs observed in participants' accounts was attributable primarily to EFAT itself or to their ongoing participation in the lifelong learning program more broadly.

Future research should examine the transferability of EFAT across different lifelong learning settings and community-based contexts, include more gender-balanced samples, employ designs that allow for differentiation between intervention-specific and contextual effects, and investigate participants' experiences over longer follow-up periods to assess the sustainability of perceived benefits.

## 5 Conclusion

The present study explored older adults' experiences of participating in EFAT, an emotion-focused psychoeducational program implemented within a University of the Third Age. The findings demonstrated that participants experienced EFAT as considerably more than an educational program. Rather, participants perceived the program as supporting emotional awareness, emotional expression, interpersonal connectedness, and the integration of emotion-regulation strategies into everyday life, while also highlighting the value of psychologically safe group environments for emotional learning and personal growth. At the same time, participants' accounts underscored the importance of recognizing the inherent limitations of group-based psycho education in addressing individualized psychological needs.

These findings contribute to the growing literature on emotion-focused psycho educational interventions by demonstrating how lifelong learning environments can function as meaningful psychosocial contexts that support emotional adaptation and psychological wellbeing in later life. By integrating emotional learning with continued education and social participation, EFAT illustrates the potential of community-based lifelong learning programs to promote healthy aging beyond traditional educational objectives.

Future research should examine the implementation of EFAT across diverse lifelong learning settings and cultural contexts, explore participants' longer-term experiences, and investigate how group-based emotion-focused psycho education may be complemented by individualized support strategies. Overall, the findings suggest that emotion-focused psycho educational programs implemented within lifelong learning environments represent a promising approach for supporting emotional wellbeing, social connectedness, and healthy aging among older adults.

## Data Availability

The raw data supporting the conclusions of this article will be made available by the authors, without undue reservation.
